# Supplementary material for: The increased production of monocytes with immunosuppressive transcriptional profiles predicts the development of SIV encephalitis
Source: bioRxiv. 2026 Oct 20:2026.10.14.759063. Preprint. [Version 1] doi: 10.64898/2026.10.14.759063 (PMC13738436; doi:10.64898/2026.10.14.759063)
Supplement: Supplementary file 1 [file NIHPP2026.10.14.759063v1-supplement-1.pdf]

bioRxiv preprint doi: https://doi.org/10.64898/2026.10.14.759063; this version posted October 20, 2026. The copyright holder for this preprint (which was not certified by peer review) is the author/funder, who has granted bioRxiv a license to display the preprint in perpetuity. It is made available under a CC-BY 4.0 International license.

Supplementary Figure

Figure S1. Transcriptomic profiling of BrdU^{+} and BrdU^{-} monocytes in SIV-associated encephalitis at necropsy. (**A**) Experimental design: SIV-infected rhesus macaques with encephalitis (SIVE) were administered BrdU before necropsy. BrdU^{+} and BrdU^{-} monocytes were sorted for RNA-seq analysis. (**B**) Principal component analysis (PCA) of BrdU^{+} and BrdU^{-} monocytes showing separation between groups (PC1 = 58.8%, PC3 = 7.7%). (**C**) Heatmap of the top 50 differentially expressed genes (DEGs) across samples. (**D**) Volcano plot of DEGs between BrdU^{+} and BrdU^{-} monocytes. Dashed lines indicate thresholds of $|\log2FC| = 1$ and adjusted $P = 0.05$. Non-significant genes are shown in grey; genes with $|\log2FC| > 1$ only in green; adjusted $P < 0.05$ only in blue; and significant genes meeting both criteria in red. (**E**) Network centrality analysis identifying hub genes associated with SIV pathogenesis based on degree, betweenness, and closeness metrics. (**F**) Sankey and dot plots showing Gene Ontology (GO) enrichment of DEGs. (**G**) Transcription factor (TF) enrichment analysis identifying key regulators using ChEA3 (ChIP-X Enrichment Analysis 3), ranked by overlap and mean rank; lower mean rank indicates higher regulatory significance.

Figure S2. Transcriptomic profiling of BrdU^{+} and BrdU^{-} monocytes in SIV infection without encephalitis at the necropsy. (**A**) Experimental design: SIV-infected rhesus macaques without encephalitis (SIVnoE) were administered BrdU prior to necropsy. BrdU^{+} (proliferating) and BrdU^{-} (non-proliferating) monocytes were sorted for RNA-seq analysis. (**B**) Principal component analysis (PCA) of BrdU^{+} and BrdU^{-} monocytes showing separation between groups (PC1 = 62.8%, PC3 = 8.4%). (**C**) Heatmap of the top 50 differentially expressed genes (DEGs) across samples. (**D**) Volcano plot of DEGs between BrdU^{+} and BrdU^{-} monocytes. Dashed lines indicate thresholds of $|\log2FC| = 1$ and adjusted $P = 0.05$. Non-significant genes are shown in

bioRxiv preprint doi: https://doi.org/10.64898/2026.10.14.759063; this version posted October 20, 2026. The copyright holder for this preprint (which was not certified by peer review) is the author/funder, who has granted bioRxiv a license to display the preprint in perpetuity. It is made available under a CC-BY 4.0 International license.

grey; genes with |log2FC| > 1 only in green; adjusted $P < 0.05$ only in blue; and significant genes meeting both criteria in red. (**E**) Network centrality analysis identifying hub genes associated with SIV pathogenesis based on degree, betweenness, and closeness metrics. (**F**) Sankey and dot plots showing Gene Ontology (GO) enrichment of DEGs. (**G**) Transcription factor (TF) enrichment analysis identifying key regulators using ChEA3 (ChIP-X Enrichment Analysis 3), ranked by overlap and mean rank; lower mean rank indicates higher regulatory significance.

Figure S3. Transcriptomic profiling of BrdU$^+$ and BrdU$^-$ monocytes during acute SIV infection. (**A**) Experimental design: SIV-infected rhesus macaques during acute infection were administered BrdU at 21-day post infection (DPI). BrdU$^+$ (proliferating) and BrdU$^-$ (non-proliferating) monocytes were sorted for RNA-seq analysis. (**B**) Principal component analysis (PCA) of BrdU$^+$ and BrdU$^-$ monocytes showing separation between groups (PC1 = 18.9%, PC2 = 11.8%). (**C**) Heatmap of the top 50 differentially expressed genes (DEGs) across samples. (**D**) Volcano plot of DEGs between BrdU$^+$ and BrdU$^-$ monocytes. Dashed lines indicate thresholds of |log2FC| = 1 and adjusted $P = 0.05$. Non-significant genes are shown in grey; genes with |log2FC| > 1 only in green; adjusted $P < 0.05$ only in blue; and significant genes meeting both criteria in red. (**E**) Network centrality analysis identifying hub genes associated with SIV pathogenesis based on degree, betweenness, and closeness metrics. (**F**) Sankey and dot plots showing Gene Ontology (GO) enrichment of DEGs. (**G**) Transcription factor (TF) enrichment analysis identifying key regulators using ChEA3 (ChIP-X Enrichment Analysis 3), ranked by overlap and mean rank; lower mean rank indicates higher regulatory significance.

Figure S4. Transcriptional reprogramming of proliferating monocytes between early and terminal SIV infection. (**A**) Experimental design: SIV-infected rhesus macaques during acute infection (at 21-day post infection (DPI)) and necropsy were administered BrdU. BrdU$^+$

bioRxiv preprint doi: https://doi.org/10.64898/2026.10.14.759063; this version posted October 20, 2026. The copyright holder for this preprint (which was not certified by peer review) is the author/funder, who has granted bioRxiv a license to display the preprint in perpetuity. It is made available under a CC-BY 4.0 International license.

(proliferating) and BrdU^{-} (non-proliferating) monocytes were sorted for RNA-seq analysis. BrdU^{+} monocytes were used to downstream analysis. (**B**) Principal component analysis (PCA) of BrdU^{+} monocytes during 21 DPI and NEC showing separation between groups (PC1 = 39.6%, PC3 = 5.7%). (**C**) Heatmap of the top 50 differentially expressed genes (DEGs) across samples. (**D**) Volcano plot of DEGs between BrdU^{+} monocytes during 21 DPI and NEC. Dashed lines indicate thresholds of $|\log2FC| = 1$ and adjusted $P = 0.05$. Non-significant genes are shown in grey; genes with $|\log2FC| > 1$ only in green; adjusted $P < 0.05$ only in blue; and significant genes meeting both criteria in red. (**E**) Network centrality analysis identifying hub genes associated with SIV pathogenesis based on degree, betweenness, and closeness metrics. (**F**) Sankey and dot plots showing Gene Ontology (GO) enrichment of DEGs. (**G**) Transcription factor (TF) enrichment analysis identifying key regulators using ChEA3 (ChIP-X Enrichment Analysis 3), ranked by overlap and mean rank; lower mean rank indicates higher regulatory significance.

Figure S5. Distinct and Shared Transcriptional Signatures in BrdU^{+} and BrdU^{-} Monocytes during SIV Infection. (A) Experimental design: BrdU^{+}/ BrdU^{-} monocytes from SIVE animals (n=3) were compared to BrdU^{+}/ BrdU^{-} monocytes from SIVnoE animals (n=4) and uninfected (no SIV) controls at necropsy (n=6). **(B)** Venn diagram showing unique and shared genes differentially expressed in BrdU^{+} SIVE monocytes/ BrdU^{-} SIVE monocytes compared to SIVnoE and control groups. **(C)** Lollipop plot of the 16 unique genes with log2FC values (all adjusted P-values<0.05). **(D)** Heatmap of 24 shared genes in BrdU^{-} monocytes at necropsy across conditions. Data represent log2FC (all adjusted P-values<0.05).

Fig S6. BrdU+ and BrdU- monocytes were sorted with extremely high purity. A) Raw data from flow cytometer demonstrating sort gating for BrdU+ monocytes B) Post-sort purity check

bioRxiv preprint doi: https://doi.org/10.64898/2026.10.14.759063; this version posted October 20, 2026. The copyright holder for this preprint (which was not certified by peer review) is the author/funder, who has granted bioRxiv a license to display the preprint in perpetuity. It is made available under a CC-BY 4.0 International license.

for the sample sorted in panel A, demonstrating purity exceeding 99.9% for the BrdU+ and BrdU- fractions.

Figure S7. Shared and stage-specific transcriptional programs of proliferating monocytes across SIV infection. (**A**) Chord diagram of differentially expressed genes (DEGs) from three comparisons (SIVE necropsy, SIVnoE necropsy, and 21 DPI), highlighting a core set of genes common to all conditions. (**B**) Heatmap of shared and unique genes across early and late stages of SIV infection. (**C**) Functional enrichment analysis of condition-specific gene sets, showing involvement in metabolism, inflammation, immune response, HIV/HAND-related pathways, and gene regulation.

Figure S8. *SMYD3* structural variants in SIVE and SIVnoE BrdU$^+$ monocytes. (**A-C**) Top 10 genes harboring SVs based on SnpEff annotation in SIVE BrdU$^+$ monocytes. (**D-F**) Top 10 genes harboring SVs based on SnpEff annotation in SIVE BrdU$^-$ monocytes.

Supplementary Tables

Table S1. Animal information used in this study.

Table S2. Shared and stage-specific transcriptional programs of proliferating monocytes across SIV infection.

Table S3. Transcriptomic profiling of BrdU$^+$ and BrdU$^-$ monocytes in SIV-associated encephalitis at necropsy.

Table S4. Transcriptomic profiling of BrdU$^+$ and BrdU$^-$ monocytes in SIV infection without encephalitis at the necropsy.

Table S5. Transcriptomic profiling of BrdU$^+$ and BrdU$^-$ monocytes during early SIV infection.

Table S6. Transcriptional reprogramming of proliferating monocytes between early and terminal SIV infection.

bioRxiv preprint doi: https://doi.org/10.64898/2026.10.14.759063; this version posted October 20, 2026. The copyright holder for this preprint (which was not certified by peer review) is the author/funder, who has granted bioRxiv a license to display the preprint in perpetuity. It is made available under a CC-BY 4.0 International license.

Table S7. Early transcriptional signatures in BrdU^{+} monocytes predict progression to SIV-associated encephalitis.

Table S8. Distinct and Shared Transcriptional Signatures in BrdU^{+} and BrdU^{-} Monocytes during SIV Infection

Table S9. Genes show significantly different mutation burdens between BrdU^{+} and BrdU^{-} cells.

Figure S1

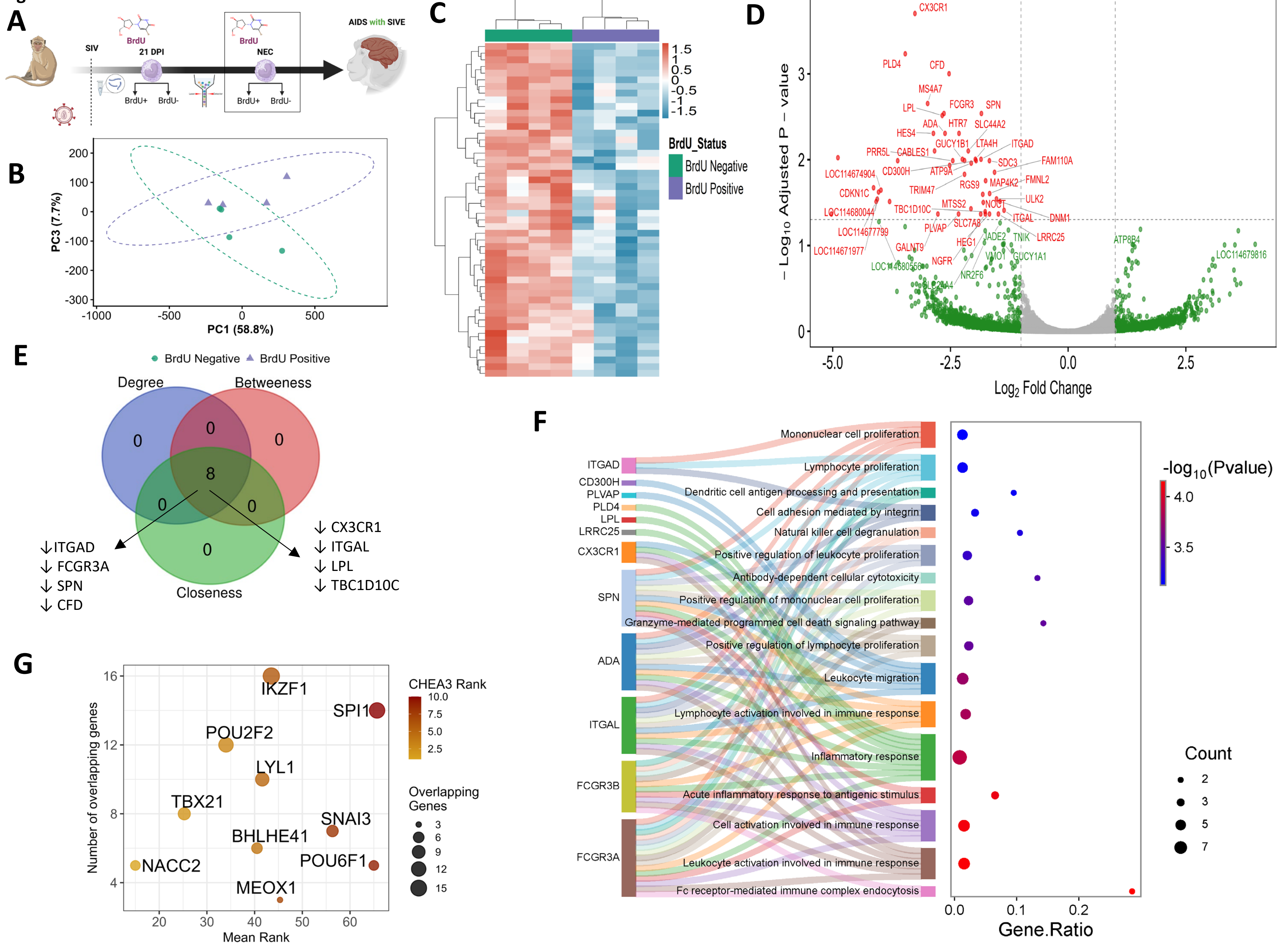
A
BrdU
SIV
21 DPI
BrdU+
BrdU-
NEC
AIDS with SIVE
B
PC3 (7.7%)
PC1 (58.8%)
BrdU Negative
BrdU Positive
C
BrdU_Status
BrdU Negative
BrdU Positive
D
− Log₁₀ Adjusted P − value
Log₂ Fold Change
CX3CR1
PLD4
CFD
MS4A7
LPL
FCGR3
SPN
ADA
HTR7
SLC44A2
HES4
GUCY1B1
LTA4H
ITGAD
PRR5L
CABLES1
SDC3
FAM110A
LOC114674904
CD300H
ATP9A
RGS9
MAP4K2
FMNL2
CDKN1C
TRIM47
ULK2
LOC114680044
TBC1D10C
MTSS2
NOCT
DNM1
ITGAL
SLC7A8
PLVAP
LOC114677799
HEG1
JADE2
TNIK
LRRC25
ATP8B4
LOC114671977
GALNT9
NGFR
VMO1
GUCY1A1
LOC114679816
LOC114680556
NR2F6
E
Degree
Betweeness
Closeness
↓ITGAD
↓ FCGR3A
↓ SPN
↓ CFD
↓ CX3CR1
↓ ITGAL
↓ LPL
↓ TBC1D10C
F
ITGAD
CD300H
PLVAP
PLD4
LPL
LRRC25
CX3CR1
SPN
ADA
ITGAL
FCGR3B
FCGR3A
Mononuclear cell proliferation
Lymphocyte proliferation
Dendritic cell antigen processing and presentation
Cell adhesion mediated by integrin
Natural killer cell degranulation
Positive regulation of leukocyte proliferation
Antibody-dependent cellular cytotoxicity
Positive regulation of mononuclear cell proliferation
Granzyme-mediated programmed cell death signaling pathway
Positive regulation of lymphocyte proliferation
Leukocyte migration
Lymphocyte activation involved in immune response
Inflammatory response
Acute inflammatory response to antigenic stimulus
Cell activation involved in immune response
Leukocyte activation involved in immune response
Fc receptor-mediated immune complex endocytosis
Gene.Ratio
-log₁₀(Pvalue)
Count
G
Number of overlapping genes
Mean Rank
IKZF1
SPI1
POU2F2
LYL1
TBX21
SNAI3
BHLHE41
POU6F1
NACC2
MEOX1
CHEA3 Rank
Overlapping Genes

Figure S2

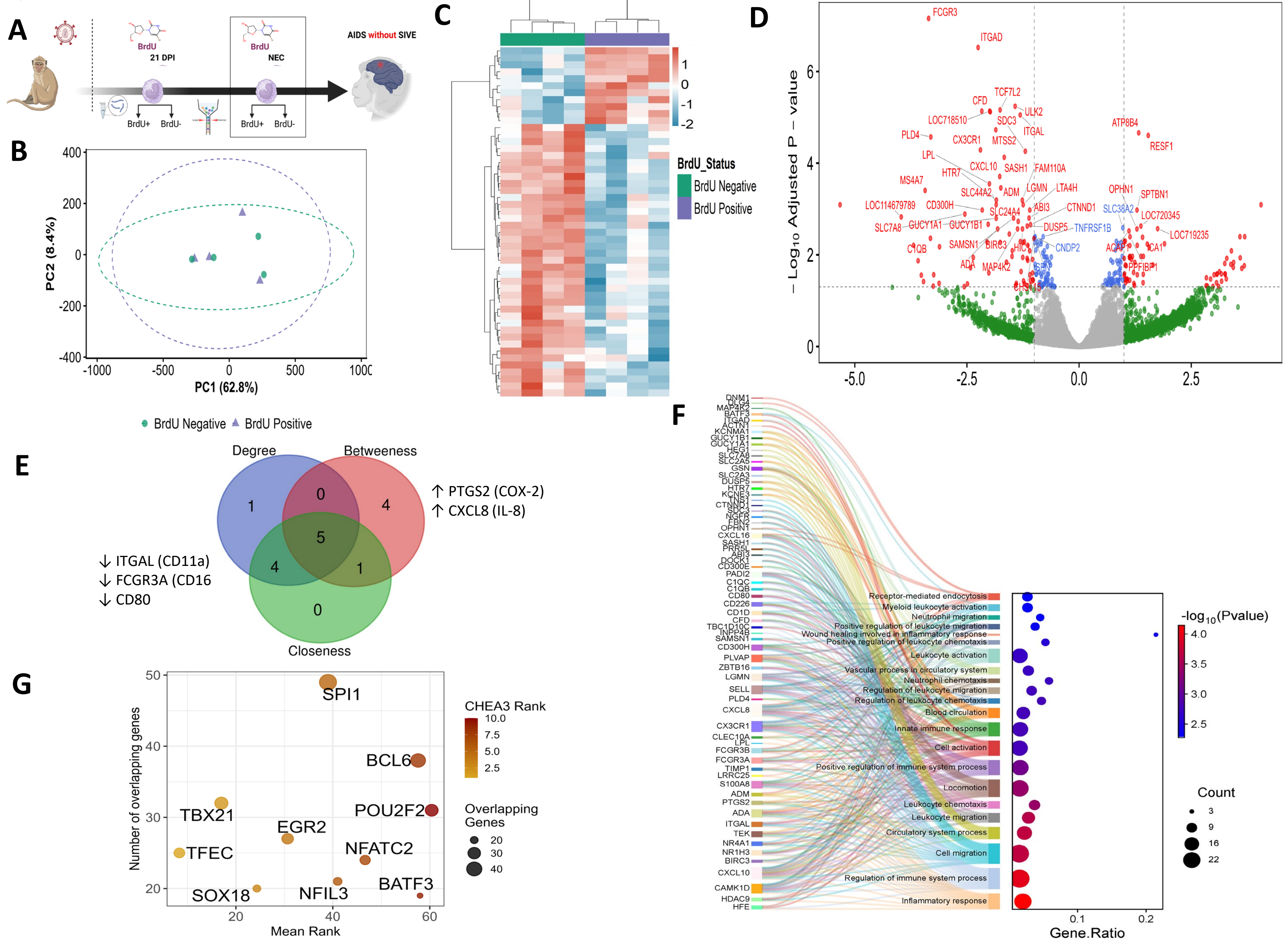
A
BrdU
21 DPI
BrdU
NEC
AIDS without SIVE
BrdU+ BrdU-
BrdU+ BrdU-
B
PC2 (8.4%)
PC1 (62.8%)
BrdU Negative
BrdU Positive
C
BrdU_Status
BrdU Negative
BrdU Positive
D
− Log₁₀ Adjusted P − value
FCGR3
ITGAD
TCF7L2
CFD
ULK2
LOC718510
SDC3
ITGAL
ATP8B4
RESF1
PLD4
CX3CR1
MTSS2
LPL
HTR7
CXCL10
SASH1
FAM110A
MS4A7
SLC44A2
ADM
LGMN
LTA4H
OPHN1
SPTBN1
LOC114679789
CD300H
SLC24A4
ABI3
CTNND1
SLC38A2
LOC720345
SLC7A8
GUCY1A1
GUCY1B1
DUSP5
TNFRSF1B
LOC719235
C1QB
SAMSN1
BIRC3
HIC1
CNDP2
ACAP1
CA1
ADA
MAP4K2
PPFIBP1
E
Degree
Betweeness
Closeness
↑ PTGS2 (COX-2)
↑ CXCL8 (IL-8)
↓ ITGAL (CD11a)
↓ FCGR3A (CD16
↓ CD80
F
Receptor-mediated endocytosis
Myeloid leukocyte activation
Neutrophil migration
Positive regulation of leukocyte migration
Wound healing involved in inflammatory response
Positive regulation of leukocyte chemotaxis
Leukocyte activation
Vascular process in circulatory system
Neutrophil chemotaxis
Regulation of leukocyte migration
Regulation of leukocyte chemotaxis
Blood circulation
Innate immune response
Cell activation
Positive regulation of immune system process
Locomotion
Leukocyte chemotaxis
Leukocyte migration
Circulatory system process
Cell migration
Regulation of immune system process
Inflammatory response
-log₁₀(Pvalue)
Count
Gene.Ratio
G
Number of overlapping genes
Mean Rank
SPI1
BCL6
TBX21
POU2F2
EGR2
TFEC
NFATC2
BATF3
SOX18
NFIL3
CHEA3 Rank
Overlapping Genes

Figure S3

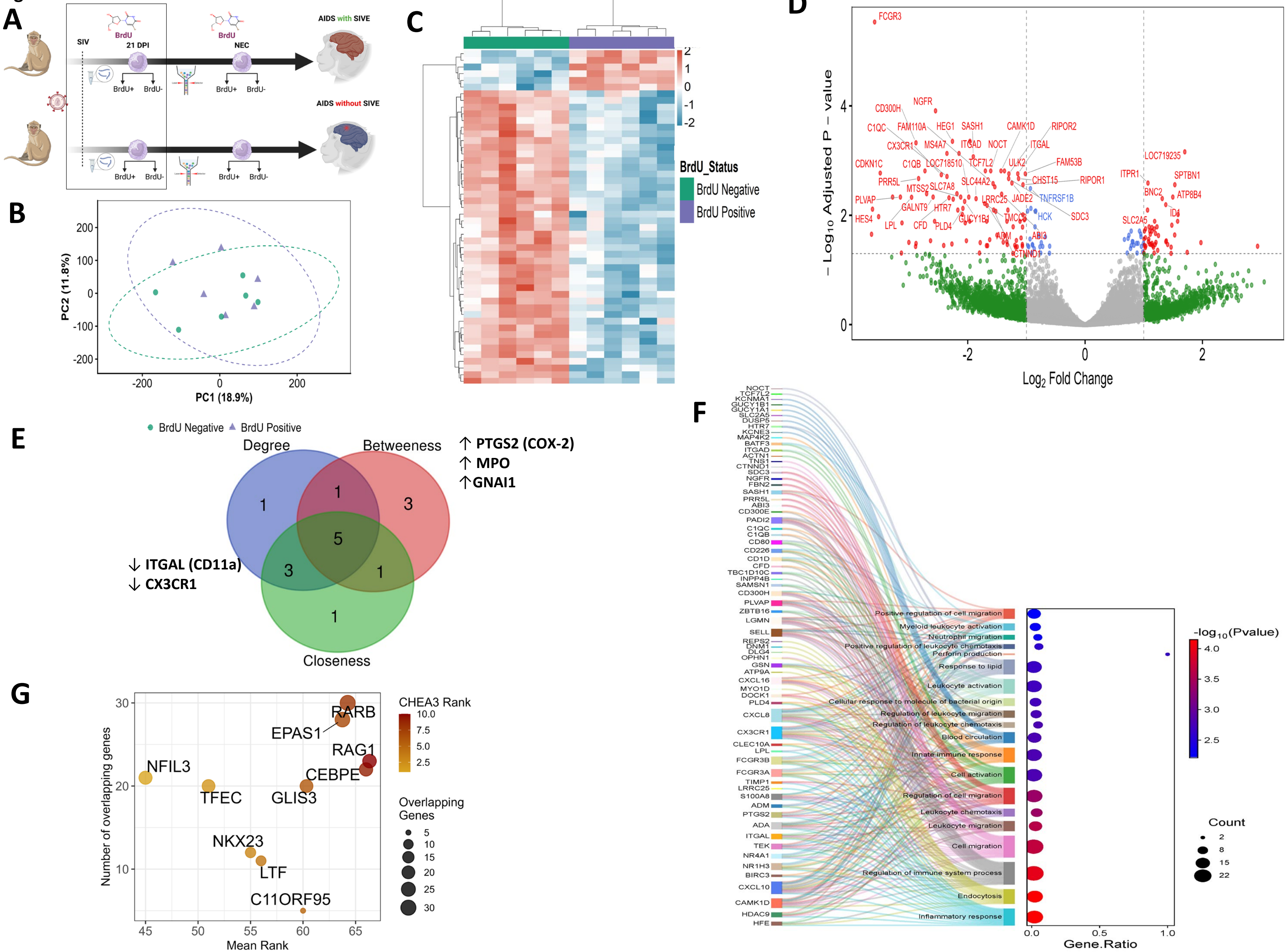

A
BrdU
21 DPI
SIV
BrdU+
BrdU-
NEC
AIDS with SIVE
AIDS without SIVE
B
PC2 (11.8%)
PC1 (18.9%)
BrdU Negative
BrdU Positive
C
BrdU_Status
BrdU Negative
BrdU Positive
D
– Log10 Adjusted P – value
Log2 Fold Change
E
Degree
Betweeness
Closeness
↑ PTGS2 (COX-2)
↑ MPO
↑ GNAI1
↓ ITGAL (CD11a)
↓ CX3CR1
F
Positive regulation of cell migration
Myeloid leukocyte activation
Neutrophil migration
Positive regulation of leukocyte chemotaxis
Perforin production
Response to lipid
Leukocyte activation
Cellular response to molecule of bacterial origin
Regulation of leukocyte migration
Regulation of leukocyte chemotaxis
Blood circulation
Innate immune response
Cell activation
Regulation of cell migration
Leukocyte chemotaxis
Leukocyte migration
Cell migration
Regulation of immune system process
Endocytosis
Inflammatory response
-log10(Pvalue)
Count
Gene.Ratio
G
CHEA3 Rank
Overlapping Genes
Number of overlapping genes
Mean Rank
RARB
EPAS1
RAG1
NFIL3
CEBPE
TFEC
GLIS3
NKX23
LTF
C11ORF95

Figure S4

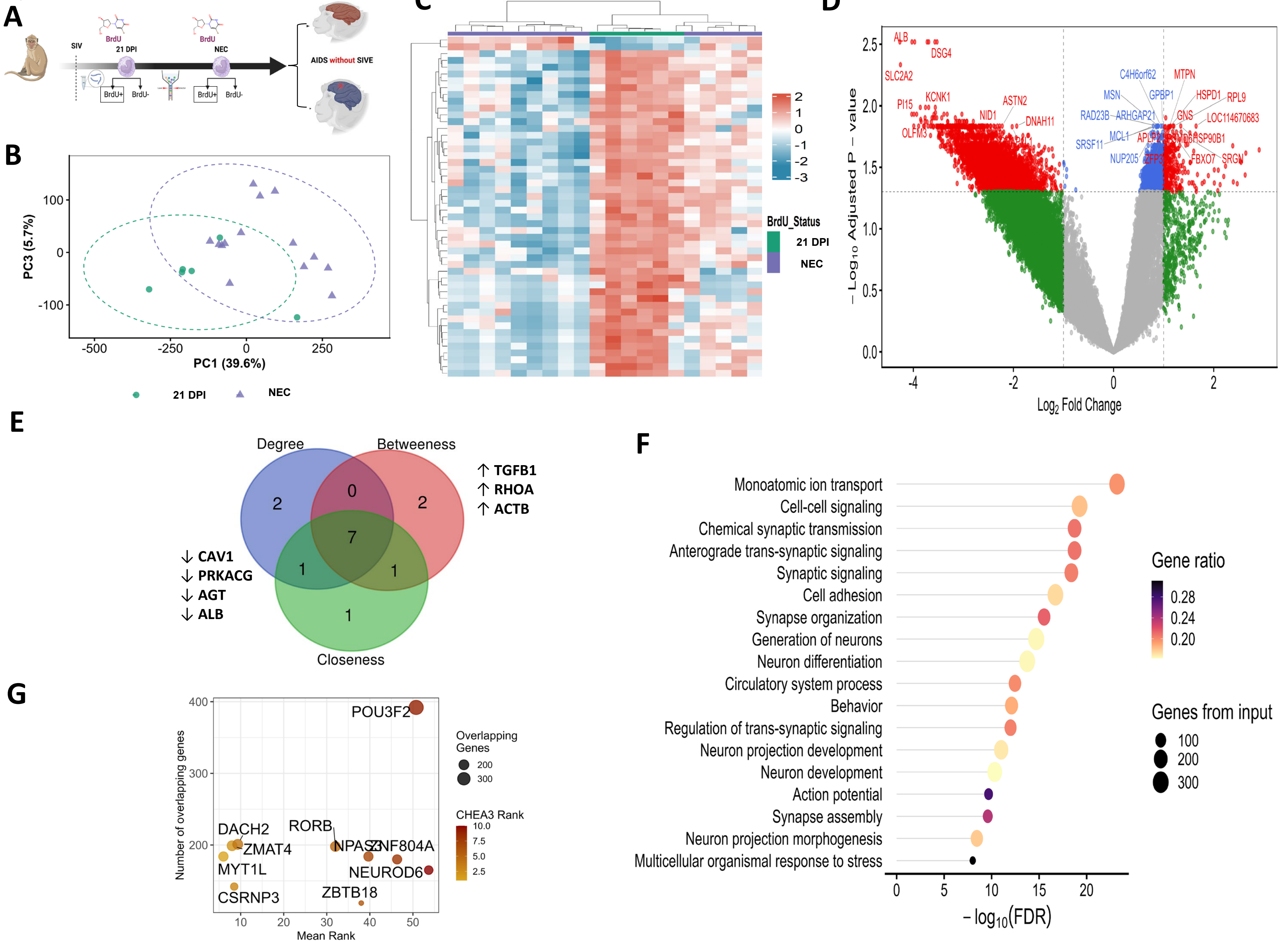
A
AIDS with SIVE
AIDS without SIVE
SIV
BrdU
21 DPI
NEC
BrdU+
BrdU-
B
PC3 (5.7%)
PC1 (39.6%)
21 DPI
NEC
C
BrdU_Status
21 DPI
NEC
D
– Log10 Adjusted P – value
Log2 Fold Change
ALB
DSG4
SLC2A2
PI15
KCNK1
OLFM3
NID1
ASTN2
DNAH11
C4H6orf62
MTPN
MSN
GPBP1
HSPD1
RPL9
RAD23B
ARHGAP21
GNS
LOC114670683
SRSF11
MCL1
NUP205
FBXO7
SRGN
E
Degree
Betweeness
Closeness
↑ TGFB1
↑ RHOA
↑ ACTB
↓ CAV1
↓ PRKACG
↓ AGT
↓ ALB
F
Monoatomic ion transport
Cell-cell signaling
Chemical synaptic transmission
Anterograde trans-synaptic signaling
Synaptic signaling
Cell adhesion
Synapse organization
Generation of neurons
Neuron differentiation
Circulatory system process
Behavior
Regulation of trans-synaptic signaling
Neuron projection development
Neuron development
Action potential
Synapse assembly
Neuron projection morphogenesis
Multicellular organismal response to stress
– log10(FDR)
Gene ratio
Genes from input
G
Number of overlapping genes
Mean Rank
POU3F2
DACH2
RORB
ZMAT4
NPAS3
ZNF804A
MYT1L
NEUROD6
ZBTB18
CSRNP3
Overlapping Genes
CHEA3 Rank

Figure S5

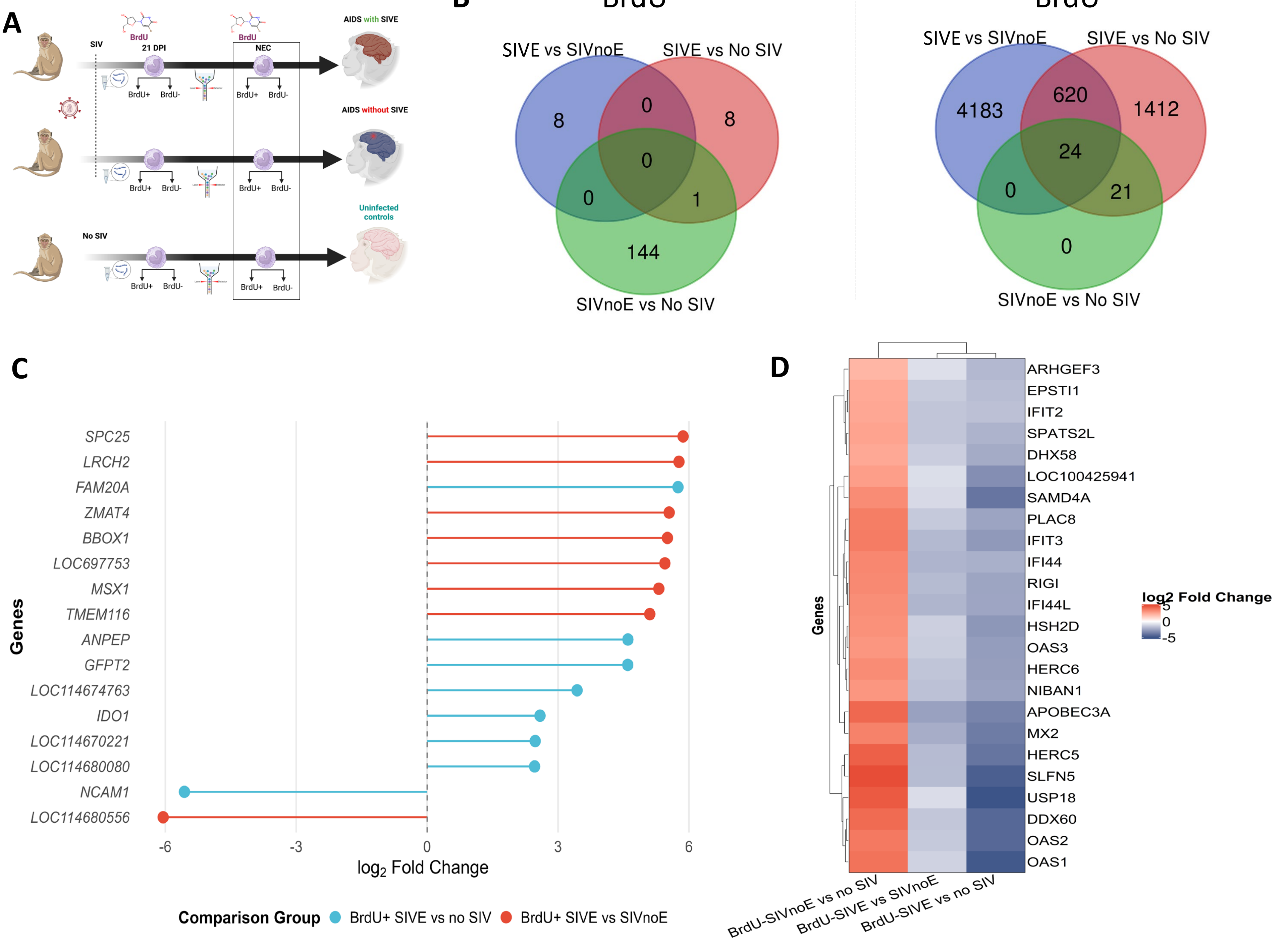
A
SIV
BrdU
21 DPI
BrdU
NEC
AIDS with SIVE
BrdU+
BrdU-
AIDS without SIVE
Uninfected controls
No SIV
B
BrdU+
SIVE vs SIVnoE
SIVE vs No SIV
SIVnoE vs No SIV
8
0
8
0
0
1
144
BrdU-
4183
620
1412
24
0
21
0
C
SPC25
LRCH2
FAM20A
ZMAT4
BBOX1
LOC697753
MSX1
TMEM116
ANPEP
GFPT2
LOC114674763
IDO1
LOC114670221
LOC114680080
NCAM1
LOC114680556
Genes
-6
-3
0
3
6
log2 Fold Change
Comparison Group
BrdU+ SIVE vs no SIV
BrdU+ SIVE vs SIVnoE
D
ARHGEF3
EPSTI1
IFIT2
SPATS2L
DHX58
LOC100425941
SAMD4A
PLAC8
IFIT3
IFI44
RIGI
IFI44L
HSH2D
OAS3
HERC6
NIBAN1
APOBEC3A
MX2
HERC5
SLFN5
USP18
DDX60
OAS2
OAS1
Genes
log2 Fold Change
5
0
-5
BrdU-SIVnoE vs no SIV
BrdU-SIVE vs SIVnoE
BrdU-SIVE vs no SIV

Figure S6

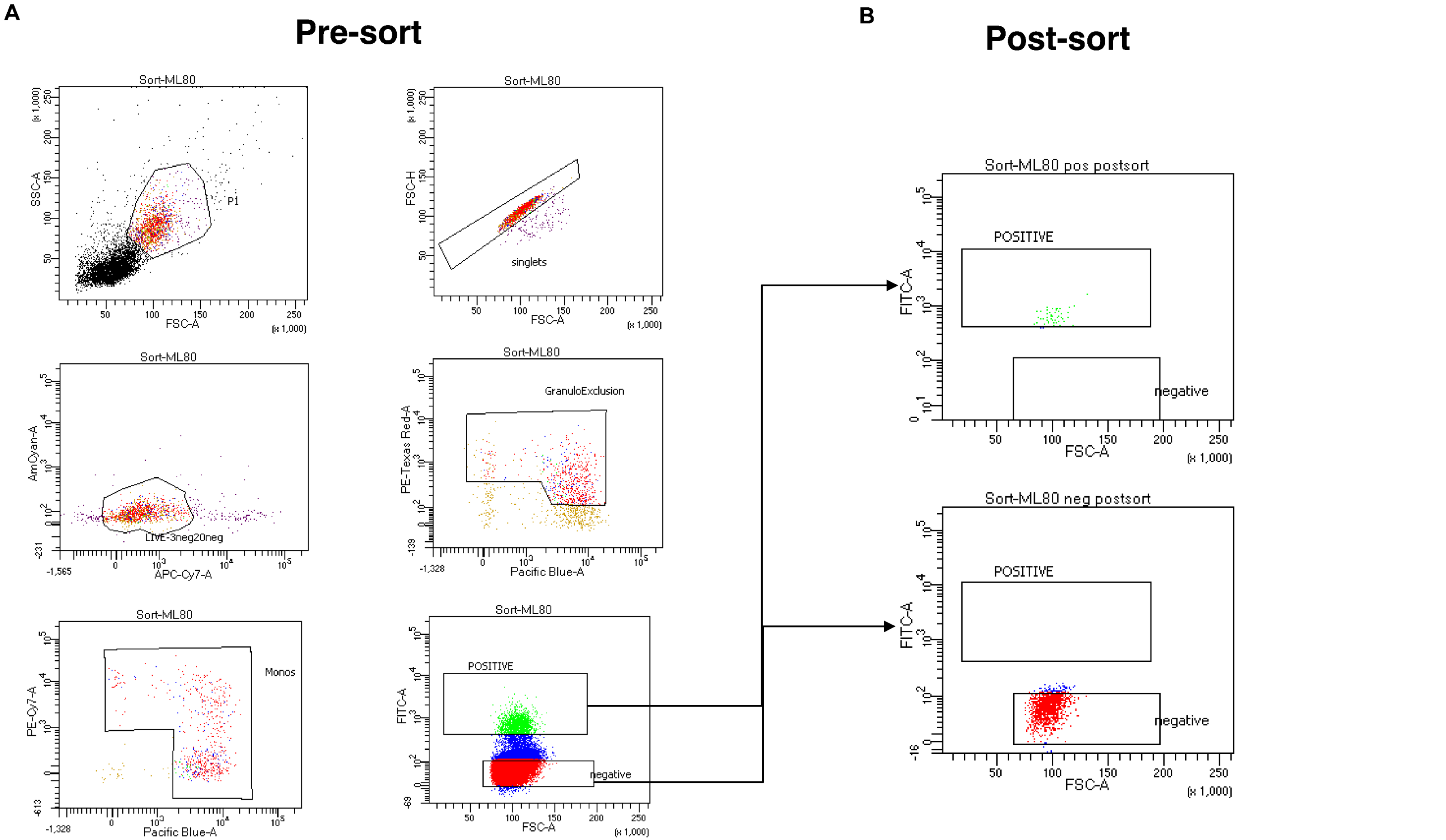

A
Pre-sort
B
Post-sort
Sort-ML80
P1
singlets
LIVE-3neg20neg
GranuloExclusion
Monos
POSITIVE
negative
Sort-ML80 pos postsort
Sort-ML80 neg postsort
SSC-A
FSC-A
FSC-H
AmCyan-A
APC-Cy7-A
PE-Texas Red-A
Pacific Blue-A
PE-Cy7-A
FITC-A

Figure S7

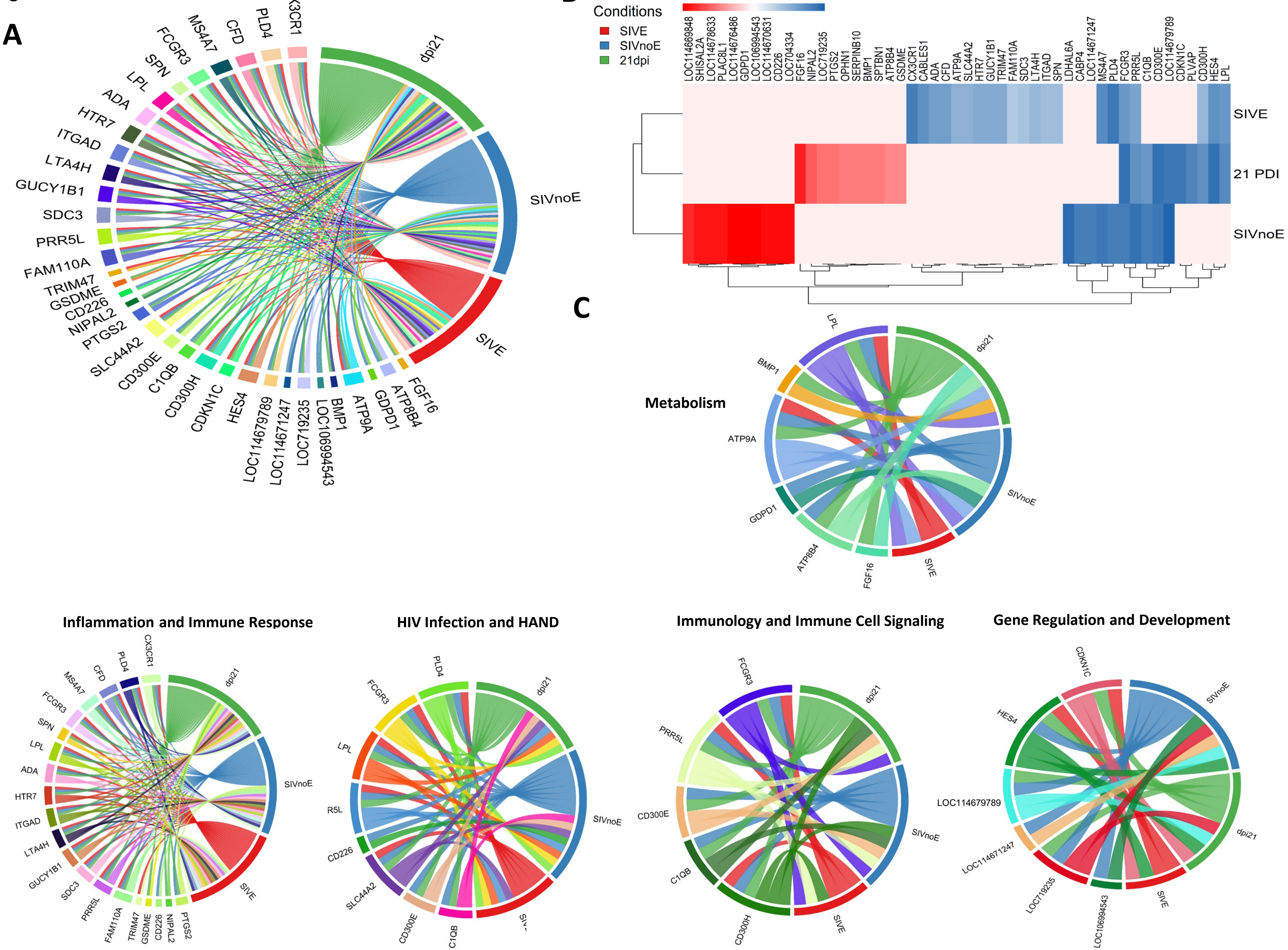

A
dpi21
SIVnoE
SIVE
CX3CR1
PLD4
CFD
MS4A7
FCGR3
SPN
LPL
ADA
HTR7
ITGAD
LTA4H
GUCY1B1
SDC3
PRR5L
FAM110A
TRIM47
GSDME
CD226
NIPAL2
PTGS2
SLC44A2
CD300E
C1QB
CD300H
CDKN1C
HES4
LOC114679789
LOC114671247
LOC719235
LOC106994543
BMP1
ATP9A
GDPD1
ATP8B4
FGF16
B
Conditions
SIVE
SIVnoE
21dpi
3 2 1 0 -1 -2 -3
LOC114669848
SHISAL2A
LOC114678633
PLAC8L1
LOC114676486
GDPD1
LOC106994543
LOC114670631
CD226
LOC704334
FGF16
NIPAL2
LOC719235
PTGS2
OPHN1
SERPINB10
BMP1
SPTBN1
ATP8B4
GSDME
CX3CR1
CABLES1
ADA
CFD
ATP9A
SLC44A2
HTR7
GUCY1B1
TRIM47
FAM110A
SDC3
LTA4H
ITGAD
SPN
LDHAL6A
CABP4
LOC114671247
MS4A7
PLD4
FCGR3
PRR5L
C1QB
CD300E
LOC114679789
CDKN1C
PLVAP
CD300H
HES4
LPL
SIVE
21 PDI
SIVnoE
C
Metabolism
LPL
BMP1
ATP9A
GDPD1
ATP8B4
FGF16
SIVE
SIVnoE
dpi21
Inflammation and Immune Response
HIV Infection and HAND
Immunology and Immune Cell Signaling
Gene Regulation and Development

Figure S8

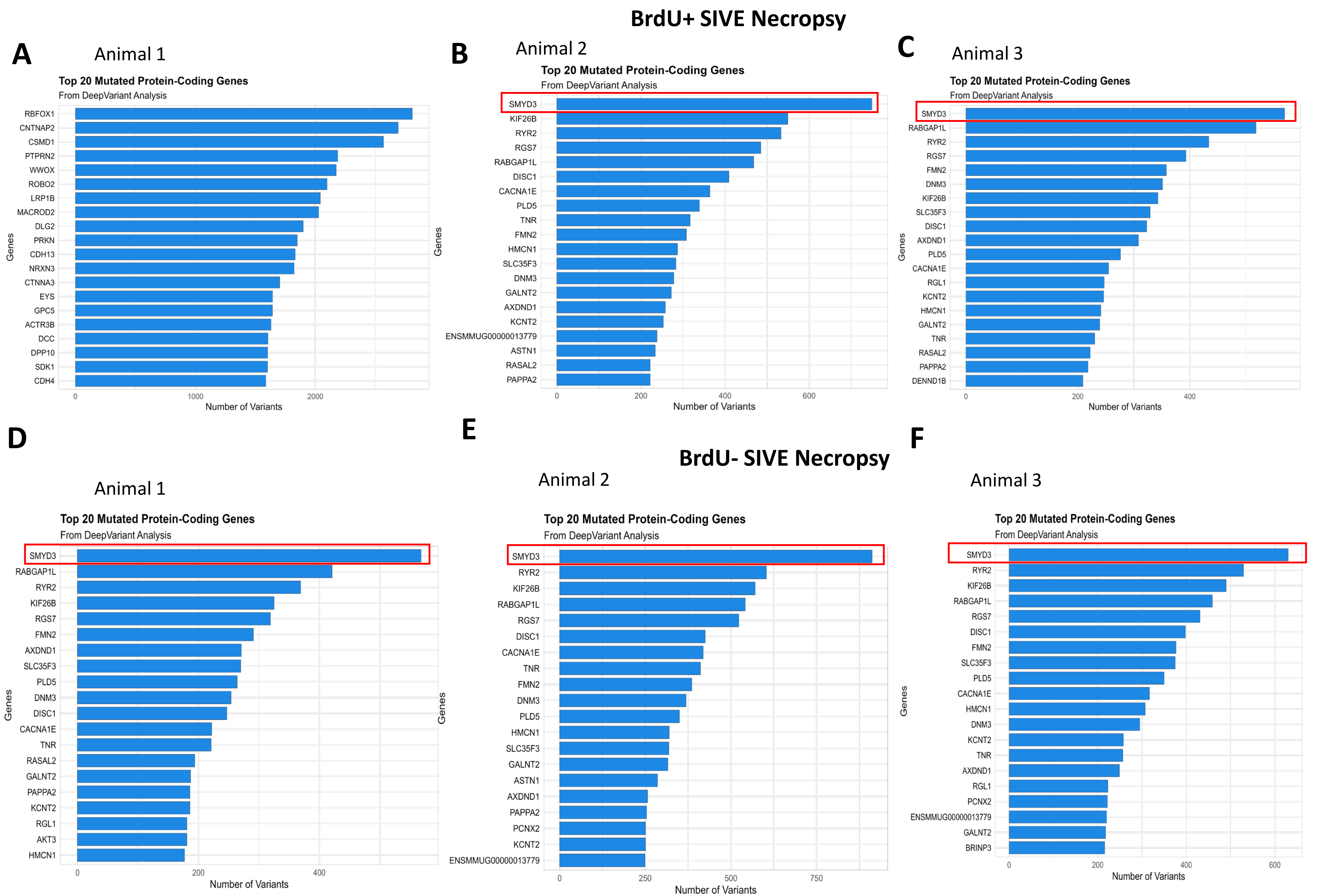
BrdU+ SIVE Necropsy
A
Animal 1
Top 20 Mutated Protein-Coding Genes
From DeepVariant Analysis
RBFOX1
CNTNAP2
CSMD1
PTPRN2
WWOX
ROBO2
LRP1B
MACROD2
DLG2
PRKN
CDH13
NRXN3
CTNNA3
EYS
GPC5
ACTR3B
DCC
DPP10
SDK1
CDH4
Genes
0
1000
2000
Number of Variants
B
Animal 2
Top 20 Mutated Protein-Coding Genes
From DeepVariant Analysis
SMYD3
KIF26B
RYR2
RGS7
RABGAP1L
DISC1
CACNA1E
PLD5
TNR
FMN2
HMCN1
SLC35F3
DNM3
GALNT2
AXDND1
KCNT2
ENSMMUG00000013779
ASTN1
RASAL2
PAPPA2
Genes
0
200
400
600
Number of Variants
C
Animal 3
Top 20 Mutated Protein-Coding Genes
From DeepVariant Analysis
SMYD3
RABGAP1L
RYR2
RGS7
FMN2
DNM3
KIF26B
SLC35F3
DISC1
AXDND1
PLD5
CACNA1E
RGL1
KCNT2
HMCN1
GALNT2
TNR
RASAL2
PAPPA2
DENND1B
Genes
0
200
400
Number of Variants
BrdU- SIVE Necropsy
D
Animal 1
Top 20 Mutated Protein-Coding Genes
From DeepVariant Analysis
SMYD3
RABGAP1L
RYR2
KIF26B
RGS7
FMN2
AXDND1
SLC35F3
PLD5
DNM3
DISC1
CACNA1E
TNR
RASAL2
GALNT2
PAPPA2
KCNT2
RGL1
AKT3
HMCN1
Genes
0
200
400
Number of Variants
E
Animal 2
Top 20 Mutated Protein-Coding Genes
From DeepVariant Analysis
SMYD3
RYR2
KIF26B
RABGAP1L
RGS7
DISC1
CACNA1E
TNR
FMN2
DNM3
PLD5
HMCN1
SLC35F3
GALNT2
ASTN1
AXDND1
PAPPA2
PCNX2
KCNT2
ENSMMUG00000013779
Genes
0
250
500
750
Number of Variants
F
Animal 3
Top 20 Mutated Protein-Coding Genes
From DeepVariant Analysis
SMYD3
RYR2
KIF26B
RABGAP1L
RGS7
DISC1
FMN2
SLC35F3
PLD5
CACNA1E
HMCN1
DNM3
KCNT2
TNR
AXDND1
RGL1
PCNX2
ENSMMUG00000013779
GALNT2
BRINP3
Genes
0
200
400
600
Number of Variants
